# Pulmonary Function Test: Relationship Between Adolescent Swimmers and Finswimmers

**DOI:** 10.7759/cureus.42711

**Published:** 2023-07-30

**Authors:** Vasileios T Stavrou, George D Vavougios, Eleni Karetsi, Zoe Daniil, Konstantinos I Gourgoulianis

**Affiliations:** 1 Laboratory of Cardio-Pulmonary Testing and Pulmonary Rehabilitation, Respiratory Medicine Department, Faculty of Medicine, University of Thessaly, Larissa, GRC; 2 Department of Neurology, Medical School, University of Cyprus, Nicosia, CYP

**Keywords:** spirometry, female, adolescent, finswimming, swimming

## Abstract

Introduction: The aim of our study was to investigate the effects of training on the static and dynamic respiratory parameters in adolescent female swimmers (SWs) and finswimmers (FSWs).

Methods: Forty-six female adolescent SWs (n=24, age=17.6±0.7 years) and FSWs (n=22, age=17.0±1.2 years) volunteered for this study. All participants underwent standard spirometry and lung volume measurements and were collected anthropometrical and morphological characteristics.

Results: The results of the groups in the pulmonary function test parameters, namely, inspiratory capacity (IC), expiratory reserve volume (ERV), and peak expiratory flow (PEF), were significantly different. Higher values of IC, ERV, and PEF were observed in the FSW group than the SW group: IC = 116.5±13.2 (SWs) vs. 125.5±11.5 (FSWs) % of predicted, p = 0.019; ERV = 121.8±14.8 (SWs) vs. 130.6±12.5 (FSWs) % of predicted, p = 0.036; PEF = 111.6±7.5 (SWs) vs. 116.3±5.0 (FSWs) % of predicted, p = 0.018.

Conclusion: The differences between groups probably reflect the activation of different muscle groups.

## Introduction

Finswimming is a competitive sports practiced at the surface and/or underwater with monofins (MF) and/or bifins (BF), with variable rigidity for propulsion purposes and snorkeling for breathing (surface and BF events) [1]. Spirometry on finswimmers (FSWs) reports higher predicted values in spirometry potentially accounting for training, the water environment, body position, and breathing pattern [1,2,3]. Breathing patterns are different during swimming and finswimming. During free swimming, the inspiratory phase of the breathing cycle is timed to coincide with arm strokes, which limit the duration of inhalation [3], while in finswimming, athletes often utilize breath holding during swimming [2]. However, breath holding during swimming (stroke cycle) and finswimming exert a physiological effect on respiratory parameters and modulate respiratory muscle strength [1], which is generally underexplored in the literature. Thus, the purpose of our study was to investigate the effects of training on the static and dynamic respiratory parameters in adolescent female swimmers (SWs) and FSWs.

## Materials and methods

Forty-six female adolescent SWs and FSWs volunteered to join this study from April 2019 to June 2019. For all athletes, the inclusion criteria were age 16-19 years, training age ≥five years, without recent injuries (>12 months) [4], training hours per week in the last two years ≥60 minutes and training ≥four times per week, and one-time participation in an official national championship in the last two years, The exclusion criteria were the presence of medical history and/or respiratory disorders (i.e., asthma) [5] and Pittsburg sleep quality index score ≥5 [6]. All SWs were competing in the previous season on 100, 200, and/or 400 m freestyle and all FSWs on 200, 400, and/or 800 m surface. The Institutional Review Board and Ethics Committee of the University of Thessaly, Greece, approved the study protocol (no. 58076/22.11.2018), and the parents of the participants provided written informed consent.

Anthropometric and morphological data were collected using Tanita MC-980 multi-frequency segmental body composition analyzer (Tanita, Netherlands). All athletes underwent standard pulmonary function test (MasterScreen-CPX, VIASYS HealthCare, Germany). For each pulmonary function test, three maximal flow-volume loops were obtained to determine the forced vital capacity (FVC), the volume that has been exhaled at the end of the first second of forced expiration (FEV_1_) and peak expiratory flow (PEF). Thoracic gas volume at the expiratory reserve volume (ERV), inspiratory capacity (IC), and vital capacity (VC) were measured, while subjects made gentle pants against the shutter at a rate of <1/s [7]. All assessments were performed at the Laboratory of Cardio-Pulmonary Testing and Pulmonary Rehabilitation (temperature: 23.1 ± 1.4 °C, humidity: 28.5± 3.1%) between 09:30 a.m. to 11:30 a.m. and after a two-day rest from training.

A power of 84% and confidence interval of 95% were adopted, with an estimated value for a type I error of 5% for the sample size calculation in this study. Twenty athletes were included in the finswimming group, and we made similar groups with the sample size, training age, and training characteristics (training hours per day and trainings per week). Data are presented as mean ± standard deviation (SD) and percentage (%). Data normality was assessed via the Kolmogorov-Smirnov one-sample test. Independent samples T-test was used to assess differences between groups. Pearson’s R correlation coefficient was used to assess continuous variables. For all the tests, a p-value of <0.05 was considered statistically significant. IBM SPSS Statistics for Windows, Version 21 (Released 2012; IBM Corp., Armonk, New York, United States) was used for all statistical analyses.

## Results

**Figure 1:**
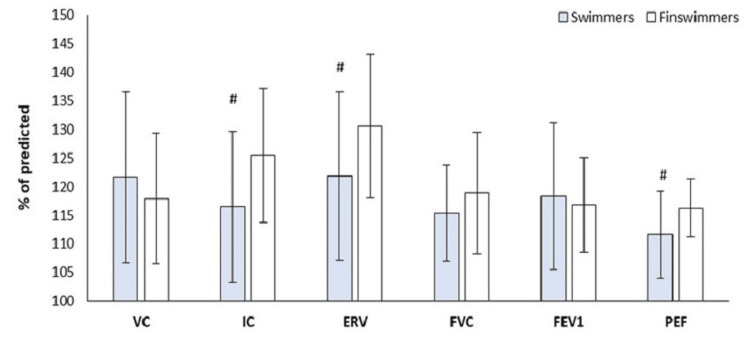
Pulmonary function test parameters between groups. ERV: expiratory reserve volume; FEV1: forced expiratory volume in the first second; FVC: forced volume vital capacity; IC: inspiratory capacity; PEF: peak expiratory flow; VC: vital capacity. # < 0.05.

Table 1 presents the athletes’ characteristics. The pulmonary function test parameters are presented in Figure 1. The variabilities FEV_1_, FVC, and VC did not show significant differences between the groups (Figure 1). However, the SW group showed higher values in FEV_1_ (118.3±12.9 vs. 116.9±8.3 % of predicted, p=0.670; percentage difference 8.8±8.7%) and VC (121.7±15.0 vs. 117.9±11.4 % of predicted, p=0.350; percentage difference 12.2±10.2%) and lower values in FVC (115.4±8.4 vs. 118.9±10.6 % of predicted, p=0.205; percentage difference 8.1±7.3%) compared to the FSW group. The variabilities IC, ERV, and PEF were significantly different between the groups (Figure 1). The FSW group showed higher values in IC (125.5±11.5 vs. 116.5±13.2 % of predicted, t_(44)_ = -2.440, p = 0.019; percentage difference 13.6±10.3%), ERV (130.6±12.5 vs. 121.8±14.8 % of predicted, t_(44)_ = -2.160, p = 0.036; percentage difference 10.9±8.1%), and PEF (116.3±5.0 vs. 111.6±7.5 % of predicted, t_(44)_ = -2.463, p = 0.018; percentage difference 6.6±5.1%) compared to the SW group. The SW group showed a statistically significant correlation between the PEF (% of predicted) and training hours per day (r=.447, p=0.028) and training days per week (r=.532, p=0.007). The FSW group showed a statistically significant correlation between training days per week and body mass index (r=.437, p=0.042), body surface area (r=.507, p=0.016), and lean body mass (r=.515, p=0.014). No significant differences were observed between the pulmonary function test parameters and anthropometrical and morphological characteristics in the groups.

**Table 1:** Athletes’ characteristics. Data are expressed as mean ± standard deviation.

		Swimmers (n=24)	Finswimmers (n=22)	P value
Age	years	17.6±0.7	17.0±1.2	0.272
Body mass	kg	65.4±9.1	60.3±8.7	0.096
Body surface area	m^2^	1.5±0.2	1.4±0.3	0.114
Lean body mass	kg	67.4±4.2	65.6±4.8	0.196
Total body water	%	50.3±3.3	51.4±2.7	0.087
Training hours per day	min	113.1±13.3	106.8±21.9	0.240
Training sessions per week	frequency	5.5±0.5	5.6±0.5	0.743

## Discussion

Our findings indicate higher values in ERV, IC, and PEF in FSWs compared to SWs. Systematic exercise increases the ERV and increases the efficiency of respiratory muscle strength of the diaphragm and intercostal muscles [8], which is caused by the intensity of exercise, increasing ventilation and lung capacity, allowing more air to move in and out of the lungs. FSWs' mandatory use of a snorkel both during training and during competitions is probably associated with systematic changes in breathing patterns that act to optimize muscle strength and increase endurance [9,10]. According to Toklu et al. [11], the use of a snorkel adds an additional dead space of 160-170 ml and causes an increase in the concentration of CO_2_ in the inspired gas due to expired air trapped in the snorkel, which is then re-inspired.

Our results show that several distinct respiratory parameters differentiate FSWs from SWs. IC values were lower in SWs than FSWs. IC refers to the volume of air that can be inspired after a normal or tidal expiration, i.e., the sum of inspiratory reserve volume and tidal volume; as such, IC is an indicator of respiratory capacity, such as during exercise, as a duration of the intensity [12]. Lower values in IC increase the likelihood of dynamic mechanical limitations at relatively low exercise intensities, thus further limiting increases in ventilation. Furthermore, IC is associated with exercise intensity and can detect exercise limitation [12] and is related to CO_2_ retention during exercise. Correspondingly, higher ERV values could reflect the effect of higher intrathoracic pressure on the diaphragm position [13]. Differences in training intensity and duration and different body structure are likely to be related to the differences observed in PEF values [14]. Moreover, PEF values were higher in FSWs compared to SWs. The PEF relates to the respiratory muscle strength and interprets the highest forced expiratory flow.

According to Vašíčková et al. [9], respiratory parameters improve more than other parameters during exercise (e.g., inhalation capacity) due to an increase in strength of the accessory respiratory muscles. The adductor respiratory muscles do not contract during full breathing and are inactive, but they are exercised during intense muscle exercises [15], leading to increased tidal volume, whereas endurance training combined with resistance training has greater effects on vital capacity and forced vital capacity.

The limitations of our study are the small sample size and the age and sex of the athletes, which could affect the different stages of puberty, biological maturation, and their physical development [1]. Moreover, the effect of menstrual cycle in pulmonary function might be a bias in our conclusions [16].

## Conclusions

Our findings revealed differences in the respiratory parameters IC, ERV, and PEF with FSWs achieving higher values than the SWs. The differences between the groups reflect probably the activation of different muscle groups, different swimming styles and body position under water, and the use of equipment. Proposals for future research will include investigating whether the parameters of cardiopulmonary exercise testing are affected by the use of snorkel for breathing during swimming.
